# Decisions in Diuretic Resistance: Oral or Subcutaneous Furosemide in Advanced Ambulatory Heart Failure?

**DOI:** 10.3390/biomedicines14092112

**Published:** 2026-09-19

**Authors:** Raquel López-Vilella, Borja Guerrero Cervera, Emilio Monte Boquet, Víctor Donoso Trenado, Julia Martínez-Solé, Luis Martínez Dolz, Luis Almenar-Bonet

**Affiliations:** 1 Heart Failure and Transplantation Unit, Hospital Universitari i Politècnic La Fe, 46026 Valencia, Spain; lopez_raqvil@gva.es (R.L.-V.); lualmenar@gmail.com (L.A.-B.); 2 Cardiology Department, Hospital Universitari i Politècnic La Fe, 46026 Valencia, Spain; martinez_luidol@gva.es; 3 Centro de Investigación Biomédica en Red de Enfermedades Cardiovasculares (CIBERCV), Instituto de Salud Carlos III, 28029 Madrid, Spain; 4 Pharmacy Department, Hospital Universitari i Politècnic La Fe, 46026 Valencia, Spain

**Keywords:** advanced heart failure, diuretic resistance, furosemide, subcutaneous administration, oral solution

## Abstract

**Objectives**: The objective of this study was to explore differences in short-term clinical response, renal outcomes, and healthcare resource utilization between two non-equivalent outpatient furosemide strategies: high-dose oral solution and subcutaneous administration. **Methods**: This retrospective observational study included 108 consecutive outpatients with advanced heart failure (HF) and diuretic resistance treated between January 2018 and December 2024. Patients received furosemide for 5 days as oral solution (250 mg/day; *n* = 81) or subcutaneous infusion via elastomeric pump (100 mg/day; *n* = 27). Primary endpoints were changes in functional status (NYHA [New York Heart Association] class), body weight, and renal function, assessed by changes in serum creatinine and estimated glomerular filtration rate and by clinically relevant worsening renal function criteria. Secondary endpoints included NT-proBNP changes and unplanned healthcare utilization at 30 days. **Results**: Both strategies were associated with short-term decongestion. Weight decreased similarly in both groups, with no difference in the proportion of patients achieving weight loss (oral 90% vs. subcutaneous 89%; *p* = 0.854). Functional improvement was more frequently observed in the oral-solution group (99% vs. 89%; *p* = 0.019). Renal outcomes were comparable between strategies, with no significant changes in eGFR and a similar proportion of patients experiencing a serum creatinine increase ≥0.3 mg/dL (25.9% vs. 22.2%; *p* = 0.85). No significant differences were observed in NT-proBNP changes. HF-related healthcare encounters decreased during the 30 days following treatment compared with the preceding 30-day period in the oral-solution group, whereas no significant within-group change was observed in the subcutaneous group. **Conclusions**: In this exploratory real-world cohort of patients with advanced HF and refractory congestion, both outpatient strategies were associated with short-term decongestion, with differences observed in symptomatic response. These findings are hypothesis-generating and warrant confirmation in prospective studies using comparable dosing strategies.

## 1. Introduction

Congestion is the most frequent and limiting clinical manifestation in patients with heart failure (HF), particularly in the advanced stages of the disease [1,2]. Its persistence despite conventional diuretic therapy is associated with worse prognosis, increased hospitalizations, and deterioration in quality of life [2].

Diuretic resistance is a complex and multifactorial phenomenon involving hemodynamic, neurohormonal, and renal adaptations that hinder sodium and water excretion despite high diuretic doses [3,4]. In patients with advanced HF, cardiorenal syndrome—present in a significant proportion of cases—further complicates congestion management [3,4,5], creating a vicious cycle that often requires more aggressive strategies such as ultrafiltration [6,7,8], which are not always accessible in the outpatient setting. Subcutaneous furosemide has been explored as an alternative strategy for outpatient management of congestion in selected patients with HF, particularly when intensification of oral diuretic therapy is insufficient or impractical [9,10]; however, this approach has relevant limitations, including local tissue alterations, device-related functional or mobility restrictions, and the need for healthcare facility visits for insertion, monitoring, and removal, particularly in frail or cachectic patients [11]. In this context, alternative strategies such as oral furosemide solution may offer practical advantages. Preliminary data from our group suggest that the oral solution furosemide can achieve comparable decongestive effectiveness with favorable clinical and analytical responses in outpatients with advanced HF and diuretic resistance [11].

This study was conceived as an exploratory comparison of two outpatient strategies used in clinical practice for the management of refractory congestion in advanced HF. Accordingly, the objective was to provide a pragmatic comparison of clinical response, renal outcomes, and healthcare resource utilization between oral furosemide solution and subcutaneous furosemide administration in outpatients with advanced HF and diuretic refractoriness.

## 2. Materials and Methods

### 2.1. Study Design

This was a single-center retrospective observational study including 108 consecutive outpatients with HF and diuretic resistance followed at a specialized HF/cardiorenal clinic between 1 January 2018 and 31 December 2024. Patients aged 18 years or older with advanced chronic HF were eligible for inclusion. Advanced HF was defined, consistent with ESC and American Heart Association criteria [1,2,12], by the presence of persistent HF symptoms despite maximally tolerated guideline-directed medical therapy, including device therapy when indicated, together with objective evidence of a structural and/or functional cardiac abnormality and recurrent or persistent congestion requiring high-dose diuretic therapy. Reduced left ventricular ejection fraction (LVEF) was not required, and patients across the spectrum of LVEF were eligible. Patients were considered suitable for outpatient intensified diuretic treatment when further ambulatory diuretic optimization was considered feasible and they remained hemodynamically stable, defined by systolic blood pressure >90 mmHg, absence of clinical signs of hypoperfusion, normal lactate levels, and preserved urine output. Patients with severe electrolyte abnormalities or deterioration in renal function requiring inpatient management were excluded from the outpatient pathway. The ability to ensure close outpatient follow-up was also required. During the 5-day treatment period, patients in both groups were contacted every 48 h by the specialized HF nurse to assess clinical evolution and treatment tolerance and were provided with direct telephone access to the specialized HF nurse to report clinical deterioration or treatment-related concerns. Clinical and laboratory parameters were reassessed at the end of treatment.

A substantial proportion of this cohort had advanced HF without eligibility for heart transplantation or durable mechanical circulatory support and was managed with a predominantly palliative/supportive strategy aimed at symptom relief, preservation of quality of life, and avoidance of hospitalization whenever clinically safe. Therefore, advanced NYHA functional class alone was not considered an indication for hospitalization in the absence of clinical criteria requiring inpatient management.

Diuretic refractoriness was defined as persistent or recurrent signs and symptoms of congestion despite high-dose oral loop diuretic therapy (≥120 mg/day), requiring further diuretic intensification and early clinical reassessment [13].

The present study represents an extension of a previously published initial single-center experience including 27 patients treated between 2018 and 2020 (17 with oral furosemide solution and 10 with subcutaneous furosemide). These patients were retained in the current cohort, which extends the inclusion period through December 2024 and incorporates 81 additional patients, representing 75% of the current cohort, treated during routine clinical practice. The rationale for the present analysis was to reassess the preliminary observations of the initial small series in a substantially larger real-world cohort.

### 2.2. Interventions

All patients received furosemide either via subcutaneous elastomeric pump or as an oral solution as part of routine clinical care. Treatment route was not assigned according to a study protocol. Instead, the choice of administration route was made during usual clinical practice, primarily according to the patient’s proximity to the clinic and ability to attend for monitoring and pump removal. The present study retrospectively analyzed these treatment decisions and their associated outcomes.

During the 5-day treatment period, the patients’ usual oral furosemide tablet regimen was temporarily discontinued and replaced by one of two standardized institutional outpatient protocols: oral furosemide solution at 250 mg/day or continuous subcutaneous furosemide at 100 mg/day via elastomeric pump.

Subcutaneous infusion group: Subcutaneous furosemide was administered through an elastomeric pump with a 240 mL capacity. The pump was prepared with 500 mg (50 mL) of furosemide and 190 mL of normal saline and standardly configured to deliver the solution at a flow rate of 2 mL/h, resulting in a furosemide dose of 100 mg/24 h. The infusion was administered subcutaneously in the subpectoral region using a Cleo (Rockford, IL, USA) 90^®^ infusion system (insertion needle, cannula with dressing, connector, and extension tubing with Luer lock), placed according to the manufacturer’s instructions. Treatment was maintained for 5 days. A total of 27 patients were treated using this strategy.

Oral solution group: Oral administration consisted of furosemide solution from ampoules at a total dose of 250 mg/day (25 mL/day), divided into two doses (12.5 mL every 12 h). Treatment was maintained for 5 days. A total of 81 patients were treated using this strategy.

Concomitant diuretic and neurohormonal therapies (thiazides, tolvaptan, acetazolamide, mineralocorticoid receptor antagonists, and sodium–glucose cotransporter 2 inhibitors [SGLT2i]) were maintained unchanged at the doses patients were receiving before the intervention throughout the 5-day treatment period. Doses of concomitant therapies are summarized in Supplementary Table S1. All patients followed a moderately sodium-restricted diet, without complete sodium restriction, in line with current HF guideline recommendations.

According to the furosemide prescribing information, the approved parenteral routes are intravenous and intramuscular; therefore, subcutaneous administration represents an off-label use, although it is described in selected palliative care guidance as an alternative in specific circumstances [9,10]. Both strategies were used as part of routine outpatient management of refractory congestion in advanced HF, based on clinical judgment and logistical feasibility. The fixed-dose regimens represented standardized local protocols and were not designed to be pharmacologically equipotent or to represent a generally accepted dose conversion between administration routes.

### 2.3. Variables and Follow-Up

Clinical, laboratory, and anthropometric variables were recorded at baseline and reassessed at the end of the 5-day treatment period.

The main effectiveness outcomes were changes in signs and symptoms of congestion, body weight, and biochemical parameters, including serum creatinine, serum sodium, and natriuretic peptide levels. Renal outcomes included changes in serum creatinine and estimated glomerular filtration rate (eGFR), as well as clinically relevant worsening renal function according to prespecified creatinine-based thresholds.

To assess symptomatic response, all patients underwent a standardized evaluation using a locally developed structured dyspnea questionnaire based on the NYHA functional classification. The questionnaire was routinely administered at baseline and at the end of the 5-day treatment period by the same specialized HF nurse, using an identical assessment procedure in both treatment groups. The nurse was aware of the treatment strategy, as blinding was not feasible in this observational setting. The complete questionnaire is provided in Supplementary Table S2.

Healthcare utilization during the 30-day follow-up included only unplanned HF-related encounters, defined as unscheduled HF clinic visits, emergency department visits, and HF-related hospital readmissions. Scheduled contacts required for treatment delivery, including placement, monitoring, or removal of the elastomeric pump, were not counted as healthcare-utilization events.

### 2.4. Ethical Considerations

Both furosemide administration strategies evaluated in this study—oral use of injectable solution and subcutaneous infusion via elastomeric pump—represent off-label applications in routine clinical practice. Given its retrospective and observational nature, no experimental intervention was introduced, and no deviation from standard clinical care occurred. The investigation conforms with the principles outlined in the “*Declaration of Helsinki*”. The research project was approved by Instituto de Investigación Sanitaria La Fe (IIS La Fe), Valencia, Spain (approval date: 6 May 2020; approval code: LAB-HID-2020-01).

### 2.5. Statistical Analysis

A descriptive analysis was performed, with continuous variables expressed as mean ± standard deviation or median with interquartile range, depending on the type of distribution (Kolmogorov–Smirnov test), and categorical variables as frequencies and percentages. Comparisons between groups were made using Student’s *t*-test for independent or paired samples, as appropriate, and Chi-square or Fisher’s exact test for categorical variables. Between-group differences in change were calculated as mean Δ(oral) − mean Δ(SC) and assessed using Welch’s *t*-test, with 95% confidence intervals (CIs). A *p*-value < 0.05 was considered statistically significant. Statistical analyses were conducted with IBM SPSS Statistics Version 27^®^ and Stata^®^ Statistics/Data Analysis 16.1. Graphs were created using PowerPoint.

## 3. Results

A total of 108 patients with advanced HF and diuretic resistance were included, of whom 81 received furosemide as an oral solution and 27 via subcutaneous pump. Baseline characteristics of both groups are shown in Table 1. Standardized mean differences indicated relatively small differences for several clinically relevant characteristics, although imbalances were observed for some covariates, most notably baseline potassium levels.

Prior diuretic treatment was generally similar between groups, with thiazides (62%), mineralocorticoid receptor antagonists (MRAs) [60%], and tolvaptan (52%) being the most frequent, and acetazolamide (12%) and chlorthalidone (8%) less commonly used. Regarding HF therapy, the most frequent treatments in both groups were beta-blockers (48%), sacubitril/valsartan (25%), and angiotensin-converting enzyme inhibitors/angiotensin receptor blockers (ACEI/ARB) (12%).

After 5 days of treatment, both groups showed favorable clinical evolution. No significant changes in NT-proBNP, serum creatinine, eGFR, urea, or sodium were observed during treatment in either group (Table 2). Serum potassium decreased significantly in the subcutaneous group (4.7 ± 0.6 to 4.3 ± 0.5 mEq/L; *p* = 0.001), while remaining stable in the oral-solution group.

**Figure 1:**
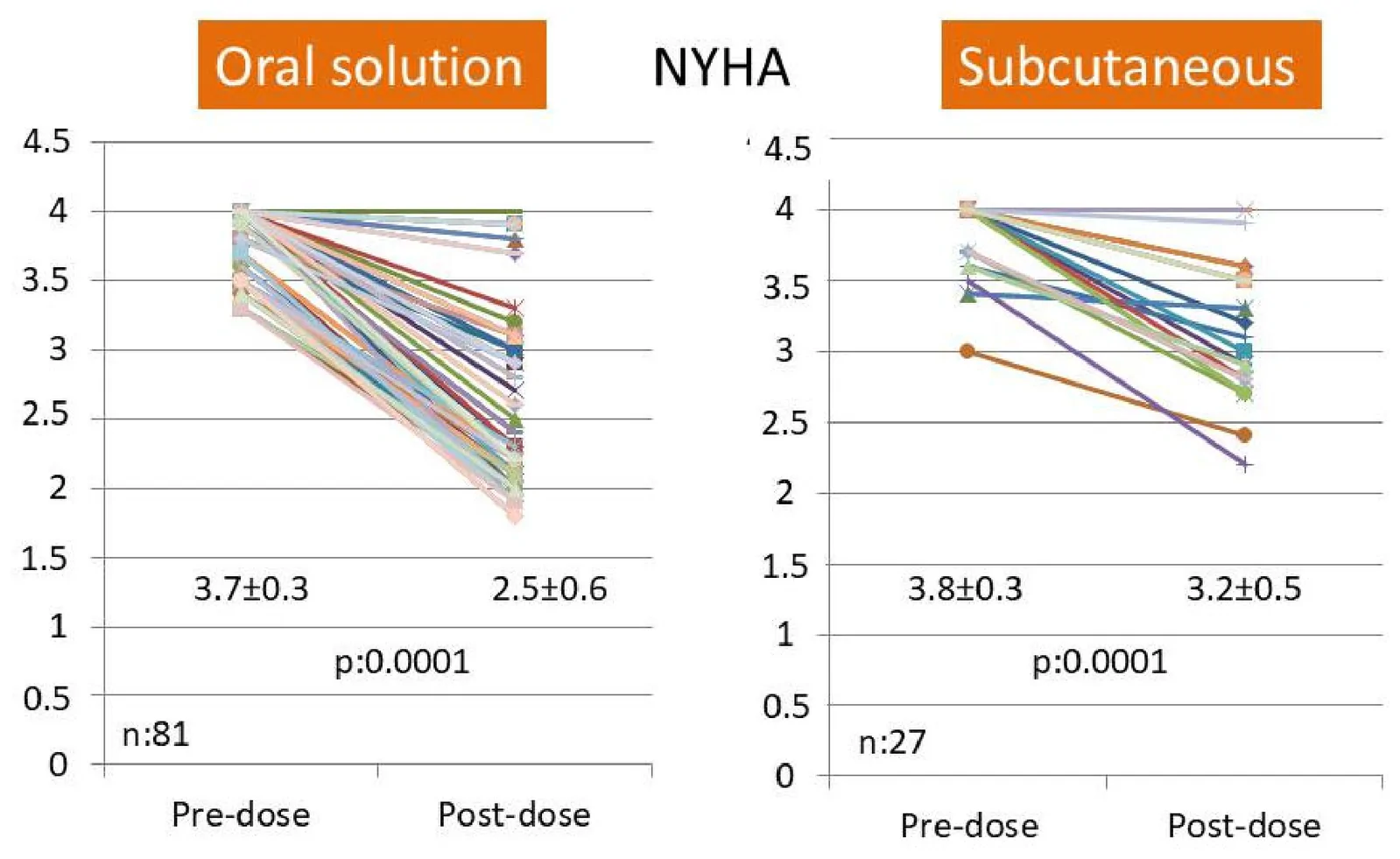
NYHA changes in both treatment groups. The different colored lines correspond to different patients.

**Figure 2:**
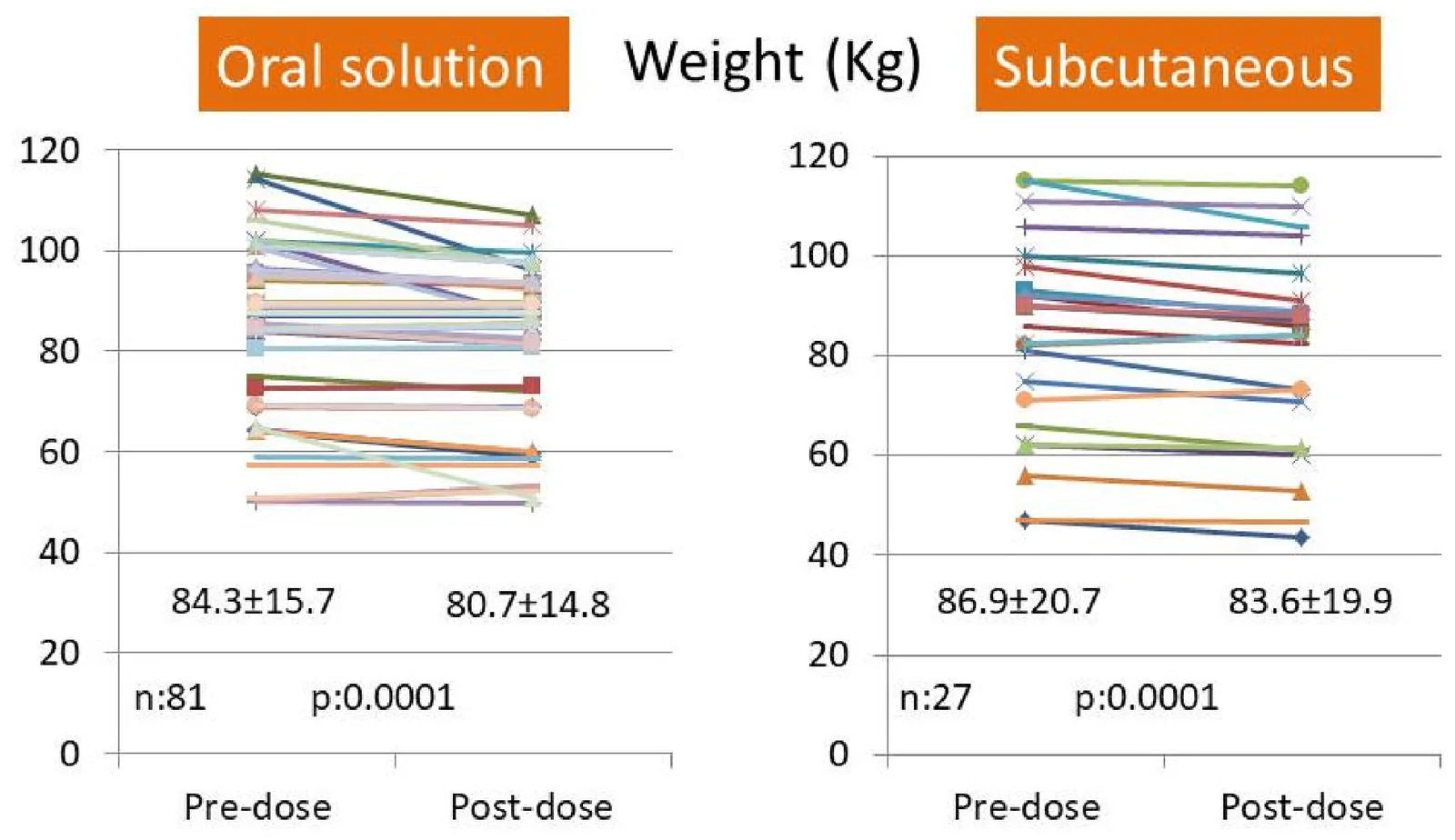
Weight in both treatment groups. The different colored lines correspond to different patients.

Functional status improved in 99% of patients receiving oral furosemide solution and 89% of those receiving subcutaneous furosemide (*p* = 0.019) (Figure 1 and Table 3). Weight decreased in both groups, and the proportion of patients experiencing weight loss was similar (90% vs. 89%; *p* = 0.854). The mean change in body weight was −3.6 kg in the oral-solution group and −3.3 kg in the subcutaneous group, with a between-group difference in change of −0.3 kg (95% CI, − 3.05 to 2.45) (Figure 2).

Although a higher proportion of patients in the subcutaneous group experienced any numerical increase in serum creatinine (63% vs. 37%; *p* = 0.019), this difference was not observed when a clinically meaningful threshold was applied. A serum creatinine increase ≥0.3 mg/dL occurred in 25.9% of patients in the oral-solution group and 22.2% in the subcutaneous group (*p* = 0.850). No significant changes in eGFR were observed in either group (oral: 46.6 ± 26.9 to 45.3 ± 19.8 mL/min/1.73 m^2^, *p* = 0.197; subcutaneous: 44.5 ± 31.9 to 45.2 ± 28.4 mL/min/1.73 m^2^, *p* = 0.117) (Table 2 and Table 3).

**Figure 3:**
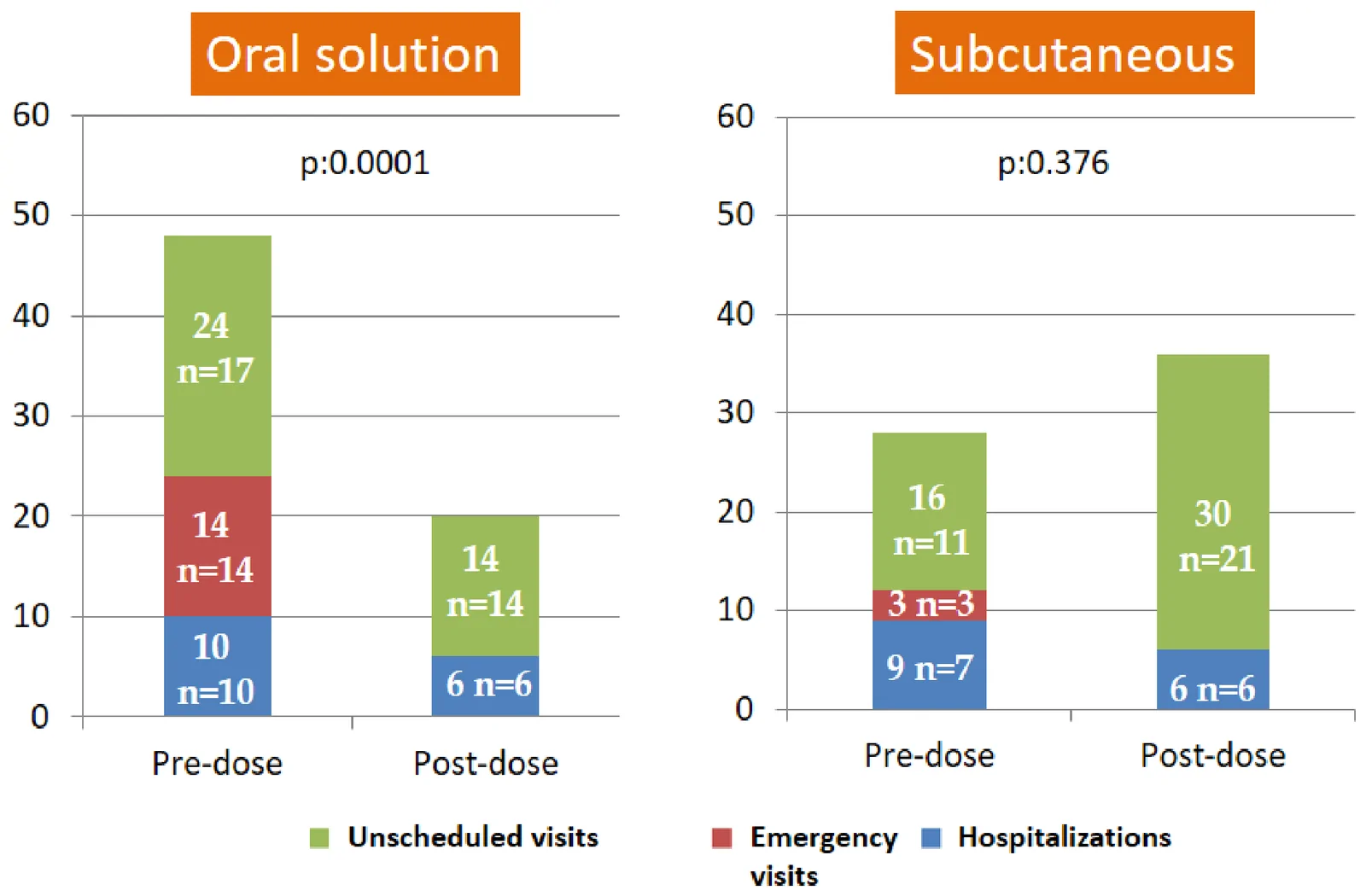
Unplanned healthcare utilization within 30 days (readmissions, Emergency Department visits, and unscheduled clinic visits). Bars represent the total number of unplanned healthcare encounters. Values above bars indicate the total number of events followed, in parentheses, by the number of individual patients experiencing ≥ 1 event (*n*). Individual patients could contribute more than one event. Scheduled contacts related to subcutaneous pump placement, monitoring, or removal were not included.

During the 30 days following treatment, unplanned HF-related healthcare encounters decreased in the oral-solution group compared with the preceding 30-day period, whereas no significant within-group change was observed in the subcutaneous group (Figure 3). In the oral-solution group, the total number of unplanned encounters decreased from 48 to 20 (*p* = 0.0001); in the subcutaneous group, encounters changed from 28 to 36 (*p* = 0.376). These analyses represent within-group comparisons and should not be interpreted as demonstrating a between-group difference in healthcare utilization. No serious adverse events or relevant complications related to either strategy were recorded.

## 4. Discussion

Congestion is the pathophysiological and clinical core of advanced heart failure (HF), driving symptoms, healthcare utilization, and prognosis; accordingly, current guidelines prioritize achieving and maintaining euvolemia [1]. Cardiorenal interactions further complicate decongestion, as venous congestion contributes to worsening renal function, while renal dysfunction perpetuates diuretic resistance and adverse outcomes [5,13,14]. Given the heterogeneous and often insufficient response to diuretics, consensus documents recommend an individualized approach, including diuretic selection, sequential escalation, and changes in the route of administration [13]. This underscores the need for pragmatic outpatient strategies, ranging from optimization of oral formulations to continuous subcutaneous administration in selected patients. Although direct comparative real-world evidence remains limited, these approaches may provide alternative strategies for achieving decongestion while allowing close monitoring of renal function [1,15]. In this study, differences in symptomatic response were observed between strategies, while HF-related healthcare encounters decreased during follow-up compared with the preceding 30-day period in the oral-solution group. These findings support the feasibility of oral furosemide solution as a practical outpatient option in selected patients.

A total of 108 patients with advanced HF and diuretic resistance were included, 81 treated with oral furosemide solution and 27 via the subcutaneous route; treatment strategy was selected primarily according to proximity to the clinic and feasibility of monitoring and pump removal. Oral furosemide is characterized by variable bioavailability, which may be further affected by intestinal congestion in patients with advanced HF [16,17]. Subcutaneous furosemide has shown bioavailability close to the intravenous route with effective diuresis and good tolerability in early-phase studies [18,19]; however, much of this evidence derives from pharmacokinetic/pharmacodynamic evaluations or modern formulations not universally available, rather than from direct real-world comparisons with oral solutions. Wearable subcutaneous delivery systems have also been described [20], but were not included in the present study.

Although several baseline characteristics were similar between groups, standardized mean differences identified residual imbalance in some covariates, particularly serum potassium. This overall clinical profile is consistent with previously described outpatient cohorts of decompensated HF treated with intravenous or subcutaneous diuretics [19,21,22]. Variability in potassium levels is expected in advanced HF due to the combined effects of MRAs, renin–angiotensin–aldosterone system (RAAS) blockade, renal dysfunction, and congestion, and both hypo- and hyperkalemia have been associated with worse prognosis, underscoring the need for close monitoring [23]. Prior diuretic therapy was similar between groups and reflected real-world use of combination diuretic strategies, with frequent use of thiazides and MRAs, in line with HFA–ESC consensus recommendations [1,12,13]. The limited use of acetazolamide early in the study period is consistent with the recent publication of the ADVOR trial and the ongoing evaluation of its role outside the acute setting [24]. The use of tolvaptan in a relevant proportion of patients aligns with its selective indication as an aquaretic, particularly in hyponatremia, despite the lack of long-term outcome benefit shown in the EVEREST trials [25,26]. The observed use of guideline-directed medical therapy should be interpreted in the context of the LVEF distribution, as only 42% of patients in the oral-solution group and 52% in the subcutaneous group had LVEF <40%, with a substantial proportion therefore having HF with mildly reduced or preserved ejection fraction. The extended inclusion period (2018–2024) should also be considered when interpreting these findings, as background HF therapy evolved substantially during this period, particularly with the progressive incorporation of SGLT2 inhibitors (SGLT2i) and optimization of guideline-directed medical therapy [27,28]. These calendar-time changes may have influenced congestion management, diuretic requirements, and clinical outcomes independently of the outpatient furosemide strategy. Although temporal effects could not be robustly assessed in the present sample, they represent a potential source of residual confounding.

After 5 days of treatment, renal function and NT-proBNP levels remained broadly stable in both groups. Although a higher proportion of patients in the subcutaneous group experienced any numerical increase in serum creatinine, this difference was not observed when a clinically meaningful threshold (≥0.3 mg/dL) was applied, and no significant changes in eGFR were observed in either group. These findings highlight the importance of interpreting small creatinine fluctuations during decongestive therapy in the context of their magnitude and accompanying changes in renal function. The reduction in serum potassium observed in the subcutaneous group was statistically significant, although mean potassium levels remained within the normal range. This finding is consistent with the potential for electrolyte changes during intensified diuretic therapy and reinforces the need for close electrolyte monitoring, as emphasized in HFA–ESC consensus documents [13]. Previous studies have supported the feasibility and generally favorable short-term tolerability of subcutaneous furosemide in selected patients with worsening HF, although changes in renal function and electrolyte disturbances, including hypokalemia, have been reported and warrant close monitoring [22,29]. The absence of a significant change in NT-proBNP over the 5-day treatment period should be interpreted in the context of the short follow-up, intra-individual biomarker variability, and the influence of renal function on natriuretic peptide concentrations. In this setting, short-term clinical assessment of congestion and diuretic response may provide more immediate information on treatment response than changes in natriuretic peptide levels [13,30]. Overall, the combination of clinical improvement and broadly stable renal and biochemical parameters is consistent with previous outpatient cohorts managed with guided diuresis, supporting the feasibility of carefully monitored ambulatory diuretic intensification in selected patients [31].

Regarding clinical response, the high rates of symptomatic improvement observed in both groups are consistent with evidence showing that intensifying diuresis in the outpatient setting—whether through subcutaneous formulations or intravenous strategies in day-hospital programs—achieves clinically significant decongestion within a few days; several programs and reviews have demonstrated symptom relief with a low event rate and good patient acceptability [32].

Weight reduction was also observed with both treatment strategies, with a similar proportion of patients experiencing weight loss. Previous studies of pH-neutral subcutaneous furosemide formulations have shown systemic exposure and diuretic/natriuretic responses approaching those of intravenous administration, together with clinical effectiveness in recent series and pilot studies [19,33]. In the present cohort, weight reduction was also frequently observed with the oral-solution strategy, supporting its potential use as an alternative approach to outpatient decongestion in selected patients.

Healthcare utilization represented the final clinical outcome evaluated in the present study. HF-related healthcare encounters decreased during the 30 days following treatment compared with the preceding 30-day period in the oral-solution group, whereas no significant within-group change was observed in the subcutaneous group. These findings are consistent with previous evidence suggesting that outpatient diuretic intensification programs may help relieve congestion and reduce acute care utilization [34,35]. However, these within-group findings should not be interpreted as demonstrating a difference in healthcare utilization between the two treatment strategies. Direct comparative data on urgent healthcare contacts between these two strategies remain scarce, with only a preliminary analysis from our group in a smaller cohort [11].

Importantly, the doses used were not equipotent (250 mg/day oral solution vs. 100 mg/day subcutaneous infusion), and differences in diuretic intensity may have influenced clinical and biochemical responses. Therefore, these findings should be interpreted as exploratory comparative-effectiveness data from routine clinical practice rather than evidence of superiority of either strategy under equipotent conditions.

An additional factor that may contribute to the heterogeneity of diuretic response in ambulatory patients with chronic HF is the development of adaptive mechanisms aimed at sodium and water conservation. Chronic exposure to loop diuretics may promote compensatory tubular sodium reabsorption and neurohormonal activation, while dietary sodium intake may further modulate net sodium balance and diuretic efficiency. Recent studies in ambulatory HF populations also suggest that adaptive, “aestivation-like” responses may influence sodium and water handling under sustained osmotic or diuretic stress. These mechanisms were not specifically assessed in our cohort, as dietary sodium intake and urinary sodium excretion were not systematically measured, but they may partly contribute to the interindividual variability in decongestive response observed in routine practice [36]. Several hypotheses could potentially contribute to the observed differences between treatment strategies, although none were directly assessed in the present study. A more gradual change in intravascular volume with oral administration could theoretically influence renal tolerance, while avoidance of a subcutaneous device might potentially reduce treatment-related discomfort and facilitate treatment acceptability. However, intravascular volume changes, patient discomfort, and treatment acceptability were not specifically measured; therefore, these mechanisms remain speculative and should be evaluated in prospective studies.

Close cardiorenal monitoring remains essential during outpatient diuretic intensification, as recommended by HFA–ESC consensus documents [13]. Rapid decongestion may induce transient intravascular hemodynamic shifts in vulnerable patients without implying sustained tubular damage, and short-term NT-proBNP changes are often modest due to biomarker kinetics and the influence of renal function; accordingly, guidelines prioritize assessment of diuretic/natriuretic response and congestion status over early natriuretic peptide changes [13,19].

From an implementation perspective, the oral-solution strategy presents fewer logistical requirements, as it does not require device placement or removal or device-related local care. Subcutaneous administration remains a useful option in selected patients but requires an organizational framework for pump placement, monitoring, and removal. Therefore, practical considerations, including patient characteristics, treatment feasibility, and local resources, should be taken into account when selecting an outpatient diuretic strategy.

This study has several limitations inherent to its retrospective, observational, and non-randomized design, including potential confounding by indication and selection bias. Treatment strategy was not assigned according to a study protocol but was determined during routine clinical care, primarily according to logistical feasibility, including proximity to the clinic and the ability to attend for monitoring and pump removal. These factors may have introduced systematic differences between groups and may also have influenced subsequent healthcare utilization, particularly outpatient and unscheduled healthcare contacts. Baseline imbalance was present in several covariates, as reflected by standardized mean differences, and could not be fully accounted for given the sample size. In addition, the non-equipotent dosing between groups (250 mg/day oral solution vs. 100 mg/day subcutaneous infusion) precludes strict pharmacological comparisons and may have contributed to differences in clinical and renal responses. The unequal group sizes reflect real-world practice but further limit direct comparisons.

Follow-up was limited to the short-term outpatient decongestion period, precluding assessment of longer-term outcomes, and objective measures of diuretic response, such as natriuresis or urine output, were not systematically available. Nutritional status and malnutrition were not systematically assessed using a standardized nutritional assessment tool; therefore, their potential influence on the response to oral furosemide, particularly in this advanced HF population, cannot be excluded.

Symptomatic response was assessed using a locally developed structured NYHA-based dyspnea questionnaire administered by the same specialized HF nurse, rather than a formally validated patient-reported outcome measure such as the KCCQ or a visual analogue dyspnea scale. Although use of the same instrument and evaluator provided a standardized assessment and avoided inter-observer variability, formal psychometric validation and reproducibility testing were not performed, and the evaluator was aware of the treatment strategy because assessments were conducted as part of routine clinical care. Given the subjective nature of this endpoint, assessment bias cannot be excluded, particularly when interpreting between-group differences in symptomatic improvement. Residual and unmeasured confounding also remain possible, and between-group differences should therefore be interpreted as exploratory associations rather than causal treatment effects.

Nevertheless, this pragmatic approach reflects routine outpatient management in advanced heart failure, where treatment decisions are guided by clinical feasibility, patient characteristics, and healthcare logistics rather than random allocation. To mitigate potential bias, consecutive patients were included, and baseline clinical and analytical characteristics were carefully compared, with residual imbalances transparently reported using standardized mean differences. Despite these limitations, the study provides novel real-world evidence in ambulatory patients with advanced heart failure and diuretic resistance using a homogeneous 5-day protocol, combined clinical and analytical assessment, and systematic evaluation of functional status by the same medical and nursing staff, thereby reducing variability. The consistency of the findings generates coherent signals that may inform clinical practice and support the design of future pragmatic randomized trials with equipotent dosing and objective measures of diuretic response.

In summary, this study addresses an evidence gap in the outpatient management of refractory congestion. Although data are available on pH-neutral subcutaneous furosemide delivered through portable devices and there is extensive experience with intravenous diuretics in day-hospital settings, to our knowledge, peer-reviewed comparative evidence on the oral use of the injectable furosemide formulation versus conventional subcutaneous infusion in adults with advanced HF and diuretic resistance remains very limited. The available literature on oral administration of injectable furosemide is scarce and fragmented, and largely derives from pediatric or compounding-focused studies rather than evaluations of clinically relevant outcomes in real-world practice.

By providing a consecutive ambulatory cohort, a homogeneous 5-day protocol, and a comparison of two strategies routinely used in our clinical practice, our study provides pragmatic real-world data on short-term clinical and renal outcomes and helps to characterize a simple, accessible, and off-label alternative used in selected clinical settings but still poorly documented in the literature.

## 5. Conclusions

In this exploratory real-world cohort of patients with advanced HF and refractory congestion, both outpatient strategies were associated with short-term decongestion, with differences observed in symptomatic response. However, because treatment allocation was non-randomized and the oral and subcutaneous regimens were not pharmacologically equivalent, these findings should not be interpreted as demonstrating superiority of one administration route over the other. Rather, they describe the outcomes associated with two different outpatient diuretic strategies as implemented in routine clinical practice and should be considered hypothesis-generating.

## Figures and Tables

**Table 1:** Description of the patients included in the study.

	Oral Furosemide (Solution), *n* = 81	SC Furosemide (Elastomeric Pump), *n* = 27	*p*	Standardized Mean Differences	Total
Age (years) *	71.3 ± 9.3	73.6 ± 8.7	0.260	0.255	72.4 ± 9.2
Male sex (*n*, %)	53 (65)	22 (81)	0.117	0.370	75 (69)
Cardiovascular risk factors (*n*, %) - Hypertension - Diabetes Mellitus - Dyslipidemia - Smoking ^1^ - Former smoker ^1^	66 (81) 36 (44) 38 (47) 11 (14) 30 (37)	18 (67) 11 (41) 16 (59) 4 (15) 11 (41)	0.109 0.737 0.267 0.872 0.731	0.343 0.075 0.249 0.035 0.076	84 (78) 47 (44) 54 (50) 15 (14) 41 (38)
Atrial fibrillation (*n*,%)	40 (49)	14 (52)	0.824	0.049	54 (50)
Time since diagnosis (years) *	10.7 ± 9.2	9.8 ± 8.4	0.342	0.102	10.1 ± 9.2
LVEF < 40% (*n*, %)	34 (42)	14 (52)	0.371	0.199	48 (44)
Reduced RVEF (*n*, %) ^2^	48 (59)	19 (70)	0.303	0.234	67 (62)
NYHA (*n*, %) - III - IV	28 (35) 53 (65)	10 (37) 17 (63)	0.816 0.816	0.052	38 (35) 70 (65)
Weight (kg) *	84.3 ± 15.7	86.9 ± 20.7	0.453	0.498	85.0 ± 17.0
Creatinine (mg/dL) *	1.9 ± 0.8	2.1 ± 0.9	0.278	0.235	2.0 ± 0.9
GFR (CKD-EPI), mL/min/1.73 m^2^	46.6 ± 26.9 (3a ^#^)	44.5 ± 31.9 (3b ^#^)	0.265	0.073	45.7 ± 27.8
Urea (mg/dL) *	113 ± 65	104 ± 59	0.467	0.145	109 ± 63
Biomarkers & Ions * - NT-proBNP (pg/mL) - Na (mEq/L) - K (mEq/L)	9465 ± 7536 141 ± 5 4.2 ± 0.6	8186 ± 7143 139 ± 6 4.7 ± 0.6	0.447 0.086 <0.001	0.174 0.362 0.833	9111 ± 8003 140 ± 6 4.4 ± 0.6
Diuretic treatment (*n*, %) - Thiazide - Acetazolamide - Tolvaptan - MRAs - Chlorthalidone - SGLT2i	53 (65) 10 (12) 40 (49) 49 (60) 6 (7) 45 (56)	14 (52) 2 (7) 16 (59) 16 (59) 3 (11) 14 (48)	0.208 0.479 0.374 0.910 0.546 0.738	0.278 0.166 0.199 0.025 0.128 0.074	67 (62) 12 (11) 56 (52) 65 (60) 9 (8) 59 (55)
Baseline oral furosemide dose (mg/day) * ^3^	170.7 ± 35.3	173.3 ± 28.3	0.850	0.081	171.7 ± 32.3
HF treatment (*n*, %) - ACEI/ARB - ARNI - Beta-blockers - Digoxin - Ivabradine	10 (12) 20 (25) 38 (47) 19 (23) 5 (6)	3 (11) 7 (26) 14 (52) 5 (19) 1 (4)	0.864 0.898 0.657 0.593 0.628	0.038 0.028 0.099 0.121 0.114	13 (12) 27 (25) 52 (48) 24 (22) 6 (6)
Potassium supplements (*n*, %)	37 (46)	12 (44)	0.911	0.025	49 (45)

* Kolmogorov–Smirnov >0.05, mean ± standard deviation. (*n*, %): number and percentage. ^1^ Smoking: current smokers within the last year; Former smokers: smoked 1–10 years prior to study. ^2^ Reduced RVEF defined as right ventricular fractional area change <35%. ^3^ Baseline furosemide dose refers to the total daily oral dose prior to initiation of the study intervention. ^#^ CKD stage is shown in parentheses according to the KDIGO GFR classification. Abbreviations: ACEI: angiotensin-converting enzyme inhibitors; ARB: angiotensin II receptor blockers; ARNI: angiotensin receptor–neprilysin inhibitor; GFR: glomerular filtration rate; HF: heart failure; K: potassium; LVEF: left ventricular ejection fraction; MRAs: mineralocorticoid receptor antagonists; RVEF: right ventricular ejection fraction; SGLT2i: sodium–glucose cotransporter 2 inhibitor; SC: subcutaneous.

**Table 2:** Analytical response in both patient groups (baseline vs. end of treatment).

	Oral Furosemide (Solution) *n*: 81			Subcutaneous Furosemide (Elastomeric Pump) *n*: 27		
	Pre	Post	*p*	Pre	Post	*p*
Urea (mg/dL) *	113 ± 65	123 ± 64	0.113	104 ± 59	105 ± 68	0.936
Creatinine (mg/dL) *	1.9 ± 0.8	1.9 ± 0.8	0.999	2.1 ± 0.9	1.8 ± 0.4	0.116
GFR (CKD-EPI) *, mL/min/1.73 m^2^	46.6 ± 26.9	45.3 ± 19.8	0.197	44.5± 31.9	45.2 ± 28.4	0.117
NT-proBNP (pg/mL) *	9465 ± 7536	9878 ± 8967	0.660	8186 ± 7143	9691 ± 9122	0.356
Na (mEq/L) *	141 ± 5	140 ± 8	0.170	139 ± 6	138 ± 9	0.518
K (mEq/L) *	4.2 ± 0.6	4.3 ± 0.7	0.174	4.7 ± 0.6	4.3 ± 0.5	0.001

* Kolmogorov–Smirnov >0.05, mean ± standard deviation. Abbreviations: K: potassium; Na: sodium; NT-proBNP: N-terminal pro-B-type natriuretic peptide; SC: subcutaneous.

**Table 3:** Proportion of patients with improvement in NYHA, weight reduction, and increases in creatinine and NT-proBNP.

	Oral Furosemide (Solution), *n* = 81	Subcutaneous Furosemide (Elastomeric Pump) *n*: 27	*p*
Improvement in functional status (NYHA) (*n*, %) *	80 (99)	24 (89)	0.019
Weight reduction (*n*, %)	73 (90)	24 (89)	0.854
Increase in creatinine (*n*, %)	30 (37)	17 (63)	0.019
% with creatinine increase ≥0.3 mg/dL	21 (25.9)	6 (22.2)	0.850
Increase in NT-proBNP (*n*, %)	20 (25)	8 (30)	0.612

(*n*, %): number and percentage. * Improvement was defined as a reduction of at least one NYHA class. Abbreviations: HF: heart failure; NT-proBNP: N-terminal pro-B-type natriuretic peptide; NYHA: New York Heart Association; SC: subcutaneous.

## Data Availability

Data are not publicly available due to institutional and patient privacy restrictions but may be obtained from the corresponding author upon request and with approval from Instituto de Investigación Sanitaria La Fe.

## Supplementary Materials

The following supporting information can be downloaded at: https://www.mdpi.com/article/10.3390/biomedicines14092112/s1, Table S1: Concomitant diuretic and neurohormonal therapy maintained during the 5-day treatment period, by administration route.; Table S2: Structured NYHA-based dyspnea questionnaire used for symptomatic assessment.

## Abbreviations

The following abbreviations are used in this manuscript:

MRAs	Mineralocorticoid receptor antagonists
HF	Heart failure
ACEI/ARB	Angiotensin-converting enzyme inhibitors/angiotensin receptor blockers
RAAS	Renin–angiotensin–aldosterone system
SGLT2i	Sodium–glucose cotransporter 2 inhibitors
LVEF	Left ventricular ejection fraction
