# Supplementary material for: Decisions in Diuretic Resistance: Oral or Subcutaneous Furosemide in Advanced Ambulatory Heart Failure?
Source: Biomedicines. 2026 Sep 19;14(9):2112. doi: 10.3390/biomedicines14092112 (PMC13604651; doi:10.3390/biomedicines14092112)
Supplement: Supplementary file 1 [file biomedicines-14-02112-s001.zip › SupplementaryTable_S1_comparativa_BORRADOR.pdf]

Supplementary Table S1. Concomitant diuretic and neurohormonal therapy maintained during the 5-day treatment period, by administration route.

Concomitant Therapy	Oral Furosemide (n=81)	SC Furosemide (n=27)	Dose, Median (Range), mg/day
Thiazide	53 (65)	14 (52)	Oral: 25 (12.5–50)<br>SC: 25 (12.5–50)
Tolvaptan	40 (49)	16 (59)	Oral: 15 (15–30)<br>SC: 30 (15–45)
Spironolactone/eplerenone	49 (60)	16 (59)	Oral: 50 (12.5–100)<br>SC: 50 (25–100)
Acetazolamide	10 (12)	2 (7)	Oral: 125 (125–125)<br>SC: 125 (125–125)
Chlorthalidone	6 (7)	3 (11)	Oral: 50<br>SC: 50
SGLT2 inhibitor	45 (56)	14 (48)	Fixed class dose

Abbreviations: SC: subcutaneous: SGLT2 inhibitor: sodium–glucose cotransporter 2 inhibitors. All concomitant therapies were continued at the same dose the patient was receiving prior to the intervention, without modification during the 5-day treatment period or during the (short) follow-up window.
