# Supplementary material for: Decisions in Diuretic Resistance: Oral or Subcutaneous Furosemide in Advanced Ambulatory Heart Failure?
Source: Biomedicines. 2026 Sep 19;14(9):2112. doi: 10.3390/biomedicines14092112 (PMC13604651; doi:10.3390/biomedicines14092112)
Supplement: Supplementary file 1 [file biomedicines-14-02112-s001.zip › SupplementaryTable_S2.pdf]

Supplementary Table S2. Structured NYHA-based dyspnea questionnaire used for symptomatic assessment.

Functional Class	Patient Profile
Class I: Absence of symptoms	No dyspnea when performing activities of daily living
Class I-II: mild symptoms with significant efforts	Dyspnea on heavy exertion (eg, climbing 3 floors)
Class II: Obvious symptoms with ordinary activity.	Mild dyspnea on moderate exertion (eg, climbing 2 floors)
Class II-III: Symptoms with moderate efforts	Significant dyspnea with moderate exertion (eg, climbing 1 floor)
Class III: Symptoms with light-moderate efforts	Dyspnea when walking (eg, walking quietly)
Class III-IV: Symptoms with light efforts	Dyspnoea at home, but can perform light household chores without breathlessness (e.g. ironing, making food…)
Class IV: Symptoms with minimal effort or symptoms at rest.	Dyspnea with light housework or at rest
